# Resuscitative transesophageal echocardiography in the emergency department: a single-centre case series

**DOI:** 10.1186/s13049-023-01077-x

**Published:** 2023-05-20

**Authors:** Fraser Kegel, Jordan Chenkin

**Affiliations:** 1 Department of Emergency Medicine, Faculty of Medicine, University of Toronto, C. David Naylor Building, 6 Queen’s Park Crescent West, Third Floor, Toronto, ON M5S 3H2 Canada; 2 Department of Emergency Medicine, Sunnybrook Health Sciences Centre, 2075 Bayview Ave., Room AG 245, Toronto, ON M4N 3M5 Canada

**Keywords:** Resuscitative transesophageal echocardiography, Resuscitation, Emergency medicine, Ultrasound, Shock, Cardiac arrest

## Abstract

**Background:**

Transesophageal echocardiography (TEE) is an emerging tool that can aid emergency physicians in treating patients in cardiac arrest and undifferentiated shock. TEE can aid in diagnosis, resuscitation, identify cardiac rhythms, guide chest compression vectors, and shorten sonographic pulse checks. This study evaluated the proportion of patients who underwent a change in their resuscitation management as a result of emergency department resuscitative TEE.

**Methods:**

This was a single-centre case series of 25 patients who underwent ED resuscitative TEE from 2015 to 2019. The objective of this study is to evaluate the feasibility and clinical impact of resuscitative TEE in critically ill patients in the emergency department. Data including changes in working diagnosis, complications, patient disposition, and survival to hospital discharge were also collected.

**Results:**

25 patients (median age 71, 40% female) underwent ED resuscitative TEE. All patients were intubated prior to probe insertion and adequate TEE views were obtained for every patient. The most common indications for resuscitative TEE were cardiac arrest (64%) and undifferentiated shock (28%). Resuscitation management changed in 76% (N = 19) and working diagnosis changed in 76% (N = 19) of patients. Ten patients died in the ED, 15 were admitted to hospital, and eight survived to hospital discharge. There were no immediate complications (0/15) and two delayed complications (2/15), both of which were minor gastrointestinal bleeding.

**Conclusions:**

The use of ED resuscitative TEE is a practical modality that provides useful diagnostic and therapeutic information for critically ill patients in the emergency department, with an excellent rate of adequate cardiac visualization, and a low complication rate.

## Background

Point of care ultrasound (POCUS) is a diagnostic modality utilized by emergency medicine physicians as a component of patient evaluations. Transesophageal echocardiography (TEE) is a valuable tool used to investigate the structures of the heart and great vessels as proximity of the esophagus to these structures makes for excellent ultrasonographic windows in real time. TEE has applications in outpatient diagnostics, critical care medicine, peri and intraoperative evaluation, and has recently been shown to be effective in emergency department resuscitations [1–3].

Emergency physicians can employ TEE to assist in the diagnosis and resuscitation of patients experiencing acute cardiovascular compromise or shock [4]. During resuscitations, TEE offers superior cardiac images compared to transthoracic echocardiography (TTE) because there are fewer structures causing impedance of ultrasound waves such as bandages, monitor stickers, and defibrillation pads [4]. Furthermore, TTE views during chest compressions are limited to the subxiphoid view. TEE can aid in the determination of the cause of a cardiac arrest and the adequacy of resuscitation efforts [4]. It can accurately identify cardiac rhythms during an arrest including cardiac standstill, ventricular fibrillation, and pulseless electrical activity and has been shown to shorten sonographic pulse checks by 10s [5, 6]. TEE can guide the vectors of chest compressions with real time feedback [5].

TEE can be used to diagnose conditions that may have precipitated shock or cardiac arrest such as aortic dissection and massive pulmonary embolism [4]. It has been shown to diagnose traumatic aortic injuries in patients who are not stable enough to undergo computed tomography [7]. TEE has even been shown to be a successful delivery method for defibrillation for ventricular fibrillation in animal subjects with high body weight [8].

Despite promising research, there are few data reviewing the outcomes of patients who undergo resuscitative TEE in the emergency department. The objective of this study is to evaluate the feasibility and clinical impact of resuscitative TEE in critically ill patients in the emergency department.

## Methods

This was a single-centre case series study that took place at an academic hospital in Toronto, Canada and was approved by our institution's Research Ethics Board (No. 3456). The ED is a regional trauma centre, the primary residency training site for emergency medicine POCUS, hosts a POCUS fellowship program, and receives over 60,000 patients annually.

Resuscitative TEE was performed by a staff emergency physician, or by a senior emergency medicine resident or POCUS fellow under direct supervision of that staff emergency physician. The emergency physician has completed advanced training in resuscitative TEE including four hours of guided practice on a high-fidelity simulator (Vimedix, CAE Healthcare), 16h of training in the operating room under the supervision of a credentialled TEE expert, and ongoing clinical practice. The emergency physician is the ultrasound fellowship director. At least one POCUS machine and an immediately accessible TEE probe were available in the ED.

The study population was a convenience sample of patients who underwent resuscitative TEE during the study period when the study physician was available in the ED. Indications for ED resuscitative TEE were cardiac arrest, shock, and post-cardiac arrest. Contraindications included an unprotected airway, a history of known esophageal surgery, esophageal varices, or other anatomic abnormalities including strictures.

TTE was considered in all patients before TEE and attempted before TEE whenever possible to obtain subxiphoid cardiac, parasternal long axis, apical four chamber, and inferior vena cava views. If TEE was performed before TTE, TTE was abandoned. TEE (Zonare P8-3TEE, Mindray Medical Systems, Shenzhen, China) views included mid-esophageal four-chamber (ME4C), mid-esophageal long axis (MELAX), trans-gastric short axis (TGSAX), mid-esophageal bicaval (MEBicaval), mid-esophageal descending aorta (MEDescAo), mid-esophageal aortic root, and mid-esophageal inflow-outflow using a standardized approach. ME4C, MELAX, TGSAX, MEBicaval, MEDescAo TEE views were obtained in a standardized fashion for each patient in the order listed. Subsequent views were obtained at the discretion of the resuscitation team and TEE operator.

The quality of each TTE and TEE view was classified as adequate, limited, or inadequate. Adequate images were defined as those with sufficienct visualization to be used for clinical decision-making purposes, as judged by the operator prospectively at the time of resuscitation. Images did not undergo a post-hoc review. POCUS and resuscitative data consisted of both categorical and written text recorded on data sheets collected prospectively at the time of resuscitation and stored in a secure location in the emergency department (“Appendix”). Pre- and post-TEE working diagnoses were recorded in real time by the resuscitation clinician and TEE operator in order to minimize recall bias.

The primary outcome of this study is a descriptive analysis of the management of critically ill patients who underwent resuscitative TEE in the ED. During the resuscitation of each patient, initial diagnoses and management were recorded in real time on data sheets before and after TEE was performed. After each TEE examination during each resuscitation, the diagnosis and management were recorded on data sheets. A change in management was reported if there was a difference between pre- and post-TEE therapeutic recommendations. Secondary outcomes included the descriptive analyses of working diagnoses, complications (immediate and delayed), patient disposition, survival to hospital discharge, and the most utilized and the quality of sonographic TEE views produced. Therapeutic recommendations were made in collaboration with the TEE operator and the resuscitation clinician. The insertion of the TEE probe was defined as easy or difficult. A difficult insertion was defined as one that required airway and/or neck manipulation, was prolonged (> 30 s) or where multiple insertion attempts were required. Probable reasons for difficult insertion and troubleshooting maneuvers were recorded prospectively during the time of resuscitation.

Immediate complications were identified and recorded at the time of resuscitation. The complete medical record for each patient who survived to hospital admission were reviewed comprehensively for any delayed complications as well as patient disposition and survival. Post-mortem reports were not reviewed for evidence of complications for patients who died in the ED. The authors assessed for complications as they have been previously defined in the literature in categories of oropharyngeal (lip laceration, loose or chipped tooth, displaced dentures, pharyngeal laceration, tongue necrosis), esophageal (odynophagia, dysphagia, laceration, perforation, Mallory Weiss tear, gastric perforation, hemorrhage), and other (splenic laceration, compression of mediastinal structures, laryngospasm, endotracheal tube displacement, airway obstruction, immediate dysrhythmias, and thermal injury or burn) [9]. Immediate complications were lip lacerations, loosened or chipped teeth, displaced dentures, pharyngeal bleeding, upper gastrointestinal bleeding, laryngospasm, endotracheal tube displacement, airway obstruction, and immediate dysrhythmias.

Descriptive statistics were used to report study outcome data. Data was collected in password protected software and analyzed using commercial software (Microsoft Excel V16.43 20110804).

## Results

During the study period 25 emergency department resuscitative TEE examinations were performed (Tables 1 and 2). The median patient age was 71 years (Range 47–95) and 10/25 patients were female. The most common indication for resuscitative TEE was cardiac arrest (16/25) followed by undifferentiated shock (7/25) and post-cardiac arrest (2/25).

**Table 1:** Patient demographics

Patients, N	25
Median age (years)	71 (Range 47–95)
Female N (%)	10 (40)
Indication for TEE, N (%)	
Cardiac arrest PEA/asystole	12 (48)
Cardiac arrest VF	4 (16)
Shock NYD	7 (28)
Post-cardiac arrest NYD	2 (8)
Disposition, N (%)	
Died in ED	10 (40)
Hospital admission	15 (60)
Survival to hospital discharge	8 (32)
Median length of stay (days)	40
TTE attempted, N (%)	12 (48)
TTE adequate view, N (%)	2 (16)

TEE, transesophageal echocardiography; PEA, pulseless electrical activity; VF, ventricular fibrillation; NYD, not yet diagnosed; ED, emergency department; TTE, transthoracic echocardiography

**Table 2:** Patients who underwent emergency department resuscitative transesophageal echocardiography

Patients N = 25		Presentation	Resuscitative TEE findings	Post resuscitative TEE diagnosis	TEE provide diagnostic clarity (19/25)	TEE influenced management (19/25)	Complications/insertion difficulty/operator Trainee N = 10, 2 complications, 3 difficult insertions Staff N = 15, no complications, 2 difficult insertions	Disposition
1	66F	Shock NYD	Global hypokinesis	Cardiogenic shock	Yes	Chrono/inotropes	None/easy/staff	ICU/survived
2	56M	Shock NYD	Hyperdynamic LV, PCE	Hypovolemic shock	Yes	Fluids	None/easy/staff	ICU/survived
3	57F	Shock NYD	Hyperdynamic LV, flat SVC	Hypovolemic shock	Yes	Fluids	None/easy/staff	ICU/survived
4	47M	Cardiac arrest PEA/asystole	RV thrombus	Pulmonary embolism	Yes	No changes	None/difficult/staff	ED/died
5	90M	Cardiac arrest VF	VF	Cardiac arrest VF	No	No changes	None/easy/trainee	ED/died
6	63F	Cardiac arrest PEA/asystole	Hyperdynamic LV	Hypovolemic shock	Yes	Fluids	None/difficult/trainee	ICU/died
7	66M	Cardiac arrest VF	Regional wall motion abnormality, PCE	Acute coronary syndrome	Yes	Cath lab	None/easy/staff	Cath lab/died
8	80F	Shock NYD	No abnormal findings	Shock NYD	No	No changes	None/difficult/staff	ICU/survived
9	66M	Cardiac arrest PEA/asystole	Cardiac standstill	Cardiac standstill	Yes	Terminate resuscitation	None/easy/staff	ED/died
10	55F	Shock NYD	Hyperdynamic LV	Hypovolemic shock	Yes	Fluids	None/easy/staff	ICU/died
11	74F	Cardiac arrest PEA/asystole	Cardiac standstill	Cardiac standstill	Yes	Terminate resuscitation	None/easy/staff	ED/died
12	86F	Cardiac arrest PEA/asystole	Right heart strain	Pulmonary embolism	Yes	Anticoagulation	UGIB^a^/easy/trainee	ICU/survived
13	87M	Cardiac arrest PEA/asystole	Inferior regional wall motion abnormality, right heart strain	Pulmonary embolism	Yes	Thrombolytics	None/easy/trainee	ED/died
14	48M	Shock NYD	Regional wall motion abnormality, flat SVC	Cardiogenic shock	Yes	Fluids Chrono/inotropes Cath lab	None/easy/trainee	Cath lab/survived
15	92M	Cardiac arrest PEA/asystole	Hypokinetic LV	Cardiac arrest NYD	No	CPR vector change Fluids Chrono/inotropes	None/easy/staff	ED/died
16	71F	Shock NYD	Hyperdynamic LV	Hypovolemic shock	Yes	Fluids	None/easy/trainee	ICU/survived
17	49M	Cardiac arrest PEA/asystole	Regional wall motion abnormality	Acute coronary syndrome	Yes	Cath lab	None/easy/staff	Cath lab/survived
18	88M	Cardiac arrest PEA/asystole	Cardiac standstill	Cardiac standstill	Yes	Terminate resuscitation	None/difficult/trainee	ED/died
19	60F	Cardiac arrest VF	Global hypokinesis	Cardiogenic shock	Yes	Chrono/inotropes	None/easy/staff	Cath lab/died
20	83F	Cardiac arrest PEA/asystole	Global hypokinesis, PCE, MS, AS, flat SVC	Cardiogenic shock	Yes	Fluids Chrono/inotropes	UGIB^b^/difficult/trainee	ICU/died
21	95M	Cardiac arrest VF	VF	Cardiac arrest VF	No	No changes	None/easy/staff	ED/died
22	62M	Post-cardiac arrest NYD	No abnormal findings	Post-cardiac arrest NYD	No	No changes	None/easy/trainee	ICU/died
23	63M	Cardiac arrest PEA/asystole	Hyperdynamic LV, RV dilated, RV thrombus	Pulmonary embolism	Yes	Thrombolytics	None/easy/staff	ED/died
24	90M	Cardiac arrest PEA/asystole	Global hypokinesis, dilated SVC	Cardiac arrest NYD	No	CPR vector change Chrono/inotropes	None/easy/trainee	ED/died
25	76M	Post-cardiac arrest NYD	Flat SVC	Post-cardiac arrest NYD	No	No changes	None/easy/staff	ICU/died

TEE, transesophageal echocardiography; F, female; M, male; NYD, not yet diagnosed; Chrono/inotropes, initiate or escalate chronotropes or inotropes; ICU, intensive care unit; LV, left ventricle; PCE, pericardial effusion; Fluids, intravenous crystalloid or packed red blood cells; SVC, superior vena cava; PEA, pulseless electrical activity; VF, ventricular fibrillation; pVT, pulseless ventricular tachycardia; RV, right ventricle; ED, emergency department; Cath lab, interventional cardiology catheterization lab; CPR, cardiopulmonary resuscitation chest compressions; MS, mitral stenosis; AS, aortic stenosis

^a^UGIB (upper gastrointestinal bleed): coffee ground fluid suctioned from the nasogastric tube on the same day as the TEE after receiving systemic anticoagulation for a pulmonary embolism, they were admitted to the ICU and started a proton pump inhibitor infusion, there was no drop in hemoglobin and did not require blood transfusion, survived to hospital discharge

^b^UGIB (upper gastrointestinal bleed): maroon coloured fluid suctioned from the nasogastric tube on the same day as the TEE, started on a PPI infusion. This patient had a history of lower gastrointestinal bleeding and was taking aspirin 81 mg daily, died in the ICU

Resuscitative TEE was performed by senior emergency medicine residents or ultrasound fellows under direct supervision in 10 cases. Probe insertion was successful in all 25 examinations (100%) with difficult insertions occurring in 9/25 (Table 5). All patients were endotracheally intubated prior to the insertion of the TEE probe.

After resuscitative TEE, the management was influenced by information gained by TEE images in 76% (N = 19) and information was diagnostically useful in 76% (N = 19) of patients (Table 3). Therapeutic recommendations included guidance of hemodynamic support with volume (8/25) or vasoactive medications (6/25), decision to transfer the patient to the cardiac catheterization lab (3/25), and decision to terminate resuscitation (3/25). The most common diagnostic contributions included hypovolemic shock (5/25), cardiogenic shock (4/25), pulmonary embolism (4/25), cardiac standstill (3/25), and acute coronary syndrome (2/25).

**Table 3:** Identified findings from transesophageal echocardiography (TEE) that provided therapeutic recommendations or diagnoses

Pre-TEE diagnoses, N (%)	
Cardiac arrest PEA/asystole	12 (48)
Shock NYD	7 (28)
Cardiac arrest VF	4 (16)
Post-cardiac arrest NYD	2 (8)
Post-TEE Diagnoses, N (%)	
Hypovolemic shock	5 (20)
Cardiogenic shock	4 (16)
Pulmonary embolism	4 (16)
Cardiac standstill	3 (12)
Acute coronary syndrome	2 (8)
Cardiac arrest NYD	2 (8)
Ventricular fibrillation	2 (8)
Post-cardiac arrest NYD	2 (8)
Shock NYD	1 (4)
Therapeutic recommendations after TEE, N (%)	
Intravenous fluids or blood products	8 (32)
Inotrope and/or chronotrope initiation/escalation	6 (24)
Cardiac catheterization lab	3 (12)
Termination of resuscitation	3 (12)
Thrombolytics or anticoagulation	3 (12)
Change chest compression vector	2 (8)

PEA, pulseless electrical activity; VF, ventricular fibrillation; NYD, not yet diagnosed; LV, left ventricle

TEE images were interpretable by operators in 100% of examinations. The most commonly obtained TEE views were the mid-esophageal four chamber (100%), mid-esophageal long axis (100%), mid-esophageal descending aorta (100%), trans-gastric short axis (96%), and mid-esophageal bicaval (68%) (Table 4). Bedside TTE was considered in all patients, was able to be performed in just under half of the patients (12/25) and yielded adequate views in two patients.

**Table 4:** Transesophageal echocardiography views

View	N attempted (%)	Adequate view N (%)
Mid-esophageal four-chamber	25 (100)	25 (100)
Mid-esophageal long axis	25 (100)	25 (100)
Mis-esophageal descending aorta	25 (100)	25 (100)
Trans-gastric short axis	24 (96)	24 (100)
Mid-esophageal aortic root	13 (52)	13 (100)
Mid-esophageal two-chamber	10 (40)	10 (100)
Mid-esophageal inflow/outflow	5 (20)	4 (80)
Mid-esophageal bicaval	17 (68)	12 (48)

10/25 patients died in the emergency department, 15/25 were admitted to hospital, and 8/15 survived to hospital discharge after a median hospital length of stay of 40 days. There were no immediate complications. There were two delayed complications that may be attributable to TEE, both of which were gastrointestinal bleeding (Table 5).

**Table 5:** Transesophageal echocardiography safety

First pass insertion, N (%)	16 (64)
Difficult insertions, N (%)	9 (36)
Challenges to insertion, N	
Ongoing chest compressions	2
Small body habitus	1
Endotracheal tube in the way	1
Reason not recorded	5
Complications, immediate, N (%)	0 (0)
Complications, delayed^a^, N (%)	
Hemorrhage	2 (13)

^a^Measured from 15 patients who survived to hospital admission

## Discussion

Resuscitative TEE is emerging as a valuable diagnostic and therapeutic tool for patients with cardiac arrest and undifferentiated shock in the ED. It is a relatively new emergency medicine modality with the first use described in the ED in 2008 [5]. It has been shown that it can be relatively easily taught to operators for resuscitations in the ED [2, 10]. There is limited published evidence on the use of ED TEE (Table 6) and this study contributes important data to the literature. In this study we found that the use of ED resuscitative TEE was associated with frequent therapeutic changes in critically ill patients. There was a low complication rate.

**Table 6:** Studies describing the utility of emergency department resuscitative transesophageal echocardiography

Study	N	Population	Complications (N)	Change in diagnostic clarity (%)	Change in management (%)	Survival to hospital discharge (%)
This study	25	Cardiac arrest, post-arrest, shock	2	76	76	32
Teran [3]	33	Out of hospital cardiac arrest	0	33	97	12
Cetena [16]	19	Out of hospital cardiac arrest	0	NR	NR	5
Jelic [1]	1	Cardiac arrest	NR	100	100	100
Fair [17]	10	ECMO guidance	NR	NR	NR	NR
Arntfield [2]	54	Cardiac arrest, shock	0	78	67	NR
Giraud [18]	1	Cardiac arrest	NR	100	0	0
Arntfield [19]	1	Cardiac arrest	NR	100	100	100
Blaivas [5]	6	Cardiac arrest, shock	NR	100	100	67

NR, not reported; ECMO, extra-corporeal membrane oxygenation

In this study, changes in management were most evident in the 12 patients who presented in cardiac arrest with non-shockable rhythms of either pulseless electrical activity (PEA) or asystole. Diagnosis was further clarified in 10/12 of these patients, resulting in management changes. Four of these patients were diagnosed with a pulmonary embolism and one with acute coronary syndrome, which prompted either anticoagulation, thrombolytics, or transfer to the cardiac catheterization lab. Two were classified as pseudo-PEA (evidence of organized cardiac activity without a detectable pulse) which necessitates different management than true PEA [11]. Therapeutic changes were also evident in the seven patients that presented with undifferentiated shock. Six of these patients were further diagnosed with either hypovolemic shock or cardiogenic shock. This differentiation is important as management often differs for patients with cardiogenic shock where initial volume resuscitation improves Frank Starling forces, but excessive volume may lead to volume overload and decreased cardiac output. The two patients diagnosed with acute coronary syndrome both presented in cardiac arrest. Electrocardiographic (ECG) changes in the immediate post-arrest period can often reflect those of ischemic changes due to cardiac hypoxia sustained during the arrest, and not necessarily from an acute coronary occlusion. TEE was helpful in these patients by demonstrating regional wall motion abnormalities in addition to the ECG findings that prompted percutaneous intervention or thrombolytics. Of the four patients diagnosed with a pulmonary embolism (PE) utilizing TEE, two had right ventricular thrombi visualized on TEE, and two had findings of new, acute right heart strain. One of the patients with new, acute right heart strain was given thrombolytics and did not achieve return of spontaneous circulation, and the other was administered anticoagulation, became hemodynamically stable enough to be taken for a CT pulmonary angiography where the diagnosis of PE was confirmed. In a similar study by Arntfield et al. of 54 ED patients, management was influenced by resuscitative TEE in 67% of patients [2]. In a 2019 study by Teran et al., ED resuscitative TEE provided therapeutic influence in 97% of cases of out of hospital cardiac arrest (N = 33) and provided diagnostic clarity in 33% [3]. Our study adds data to a limited body of literature that diagnosis and management of acutely ill patients in the ED can be influenced by TEE examinations.

Arrest and peri-arrest clinical data can be often obtained by utilization of TTE, however, TEE has expanded scope (better ability to visualise regional wall motion abnormalities and pulmonary emboli, and improve chest compression vectors), the benefit of providing images without pausing chest compressions, and allowing the resuscitation team to monitor the effects of treatment in real time. The authors propose that resuscitative TEE should be considered an element of care for patients presenting to the ED with cardiac arrest and undifferentiated shock in centres with access to TEE.

In this study, TTE images were obtained 48% of patients, with adequate views achieved in 16% of patients, which is consistent with previous literature for critically ill patients [12]. This was due to mechanical factors including obstructing defibrillator pads, cardiac monitors, and ongoing operator CPR, patient factors including body habitus, clinical factors where the area under investigation was unable to be assessed adequately by TTE (thoracic aorta), and abandonment of TTE for TEE. Many TTE images are obtained during cardiac arrest during the pulse and rhythm checks, which are limited to less than 10s.

Our study utilized a variety of combinations of eight standard TEE views for each patient but relied most heavily on five views (Table 3). Operators were able to generate adequate views in every patient. The American College of Emergency Physicians has recommended using a three-view approach during resuscitation (mid-esophageal 4-chamber, mid-esophageal long-axis, and trans-gastric short-axis views) [15] while other literature recommends including the mid-esophageal bicaval view as well [2]. At the time of our study inception there were limited recommendations on which TEE views were most beneficial during ED resuscitative TEE so varying combinations of eight views were investigated.

There is extensive literature discussing the complication rates for elective, surgical, and intensive care unit (ICU) TEEs. There is very limited published evidence discussing the safety of ED resuscitative TEE, with complication rates ranging from 0 to 12.6% [2, 3, 9, 13, 14]. In our study there were no immediate complications. Delayed complications that may have been attributable to TEE were identified in two of the 15 patients who survived to hospital admission. One patient presented in cardiac arrest, was diagnosed with a pulmonary embolism using TEE, was treated, admitted to the ICU, and survived. This patient experienced coffee ground fluid suctioned from the nasogastric tube (NGT) on the same day as the TEE after receiving systemic anticoagulation for a pulmonary embolism. This was treated with a proton pump inhibitor (PPI) infusion. The second patient presented in cardiac arrest, was further diagnosed with cardiogenic shock and survived to ICU. This patient experienced maroon coloured fluid suctioned from the NGT on the same day as the TEE, was also started on a PPI infusion, and died in the ICU after refractory ventricular fibrillation five days later. This patient had a history of lower gastrointestinal bleeding and was taking aspirin 81 mg daily. Neither patient had a significant drop in their hemoglobin nor required blood products. This study was not powered for a safety analysis.

Our data should be interpreted in light of several limitations. Although resuscitation and primary outcome data was obtained prospectively, this study is limited by the retrospective nature of the medical record review for each patient to collect data for secondary outcomes. For the 16 patients who presented with cardiac arrest, pre-hospital data including down time and out of hospital resuscitative data were not available. Our sample size is relatively small. Although there were frequent changes in management after resuscitative TEE, this study was not powered to detect a significant change in outcome for these patients. There was one operator who was trained in TEE at the study site and as a result, consecutive patients who met the indications for ED resuscitative TEE could not be recruited, and a convenience sample was obtained. The TEE scans were all performed or supervised by one staff emergency physician, which decreases interrater variability but limits the generalizability of the results.

## Conclusion

In this study, we found that the use of resuscitative TEE was associated with frequent changes in management for critically ill patients in the ED. In addition, the use of TEE resulted in a high rate of adequate cardiac visualization with a low complication rate. Larger prospective randomized studies are needed to determine the impact of resuscitative TEE on patient outcomes in the ED.

## Data Availability

The authors will make available the data and materials of this study.

## Abbreviations

TEE: Transesophageal echocardiography
ED: Emergency department
POCUS: Point of care ultrasound
TTE: Transthoracic echocardiography
ME4C: Mid-esophageal 4 chamber
MELAX: Mid-esophageal long axis
TGSAX: Trans-gastric short axis
MEBicaval: Mid-esophageal bicaval
MeDescAo: Mid-esophageal descending aorta
PEA: Pulseless electrical activity
ECG: Electrocardiography
NGT: Nasogastric tube
PPI: Proton pump inhibitor
ICU: Intensive care unit
